# A recombinant multi-antigen vaccine with broad protection potential against avian pathogenic *Escherichia coli*

**DOI:** 10.1371/journal.pone.0183929

**Published:** 2017-08-24

**Authors:** Angelica Van Goor, Zachary R. Stromberg, Melha Mellata

**Affiliations:** Department of Food Science and Human Nutrition, Iowa State University, Ames, Iowa, United States of America; Cornell University, UNITED STATES

## Abstract

Chickens are a major source of protein worldwide, yet infectious diseases continue to threaten the poultry industry. Avian pathogenic *Escherichia coli* (APEC), a subgroup of extraintestinal pathogenic *E*. *coli* (ExPEC), causes colibacillosis in chickens resulting in economic loss because of treatment, condemnation of products, and death. In this study, we evaluated a recombinant antigens (rAg) vaccine combining common ExPEC surface proteins EtsC, OmpA, OmpT, and TraT for broad protective potential against APEC infections in chickens. The specific objectives were to evaluate antibody (serum) and cytokines (lymphoid organs) responses to vaccination; *in vitro* bactericidal ability of serum and splenocytes against multiple APEC serotypes; and *in vivo* protection against APEC challenge in chickens. Groups of four-day old chickens (N = 10) were vaccinated twice (two-week interval) subcutaneously with rAgs alone or in combination and CpG adjuvant or PBS (control). IgY antibody in the serum and mRNA expression of IL-1β, IL-6, IL-18, IFN-γ, IL-4, IFN-β, and IL-8 in bursa, spleen, and thymus were measured using ELISA and RT-qPCR, respectively. Serum and splenocytes were tested for their bactericidal ability *in vitro* against multiple APEC isolates. Vaccinated and non-vaccinated chickens were challenged with 10^8^ CFU of APEC-O2 via air sac at 31 days post first vaccination. Vaccine protection was determined by the decrease of bacterial loads in blood and organs (lung, heart, spleen, and liver), as well as gross colibacillosis lesion scores in air sac, heart, and liver. Vaccination significantly (*P* < 0.05) elicited IgY against specific antigens, induced immune related mRNA expression in the spleen and bursa, reduced *in vitro* growth of multiple APEC serotypes, and decreased bacterial loads in the heart and spleen, and gross lesion scores of the air sac, heart and liver in chickens. The vaccine reported may be used to provide broad protection against APEC strains, increasing animal welfare and food production.

## Introduction

The global population is expected to reach over 9 billion by 2050 [1]. It is estimated that overall food production must increase by over 70% to feed the growing population with the same amount of land and water available today [2], and the demand for poultry is projected to surpass all other meat types [3]. Extraintestinal pathogenic *Escherichia coli* (ExPEC) are a concern for human and animal health causing a variety of extraintestinal diseases. Avian pathogenic *E*. *coli* (APEC), a subgroup of ExPEC, typically inhabits the intestinal tract of poultry species, but when in extraintestinal sites of the host can cause a wide range of diseases resulting in economic losses to the poultry industry [4–6]. APEC cause colibacillosis in poultry, characterized by systemic inflammation, invasion of organs especially the lung, heart, liver, and spleen where it causes gross lesions that may result in condemnation of product, and sometimes death. With increased regulation on use of antibiotics to prevent infections and antibiotic resistance in bacteria, especially *E*. *coli*, alternatives are needed to improve animal health and welfare, such as new vaccines. Serotype-specific vaccine strategies to prevent APEC infections in chickens have been widely unsuccessful, likely because a wide range of APEC serogroups can infect poultry flocks, including O1, O2, O18, O55, and O78 [4, 7]. Additionally, APEC shares characteristics of ExPEC including adhesins, iron uptake systems, and protectins/invasins, it has potential to be a human pathogen, and therefore these strains potentially pose a threat to food safety [8–10].

Bacterial surface antigens, such as outer membrane proteins (OMPs), are typically good vaccine antigens against enteric bacteria, because they are more accessible to uptake and processing by antigen-presenting cells. A vaccine containing multiple surface common ExPEC proteins has potential to provide broad protection. Previously, the use of recombinant antigens (rAg) of *E*. *coli* adhesins (EcpA and EcpD) and iron-uptake (IutA and IroN) in a vaccine has been effective against ExPEC in a mouse sepsis model using both active and passive immunization [11].

Here we describe a novel vaccine where four surface proteins were combined, i.e., EtsC, OmpA, OmpT, and TraT. We chose these surface antigens based on their high prevalence in field isolates of APEC, as reported either here and by others. As well as the implications of some of them in virulence and/or association to plasmids. The OMPs function in stabilization of the cellular membrane of bacteria. EtsC is the outer membrane protein of a putative Type 1 secretion system [12]. OmpA that functions as a physical linkage between the outer membrane and peptidoglycan [13] contributes to virulence of *E*. *coli* via increased serum resistance mechanism [14] and cell invasion, as well as involvement in host immune activation and antimicrobial resistance [15]. In neonatal meningitis *E*. *coli* (NMEC), OmpA is critical for bacterial adhesion to brain microvascular endothelial cells implicating its importance in meningitis [16]. The OmpT protein may have a role in adherence to eukaryotic cells [17], and may be implicated in NMEC virulence because it is highly regulated in human brain microvascular endothelial cells [18]. OmpT is important to the pathogenicity of APEC, and has a role in adherence and invasion of host cells [19]. Lastly, TraT is encoded by large plasmids, typically F-like conjugative plasmids and has functions in plasmid transfer [20] and serum resistance [21, 22].

The goal of this study was to evaluate EtsC, OmpA, OmpT, and TraT proteins for their antigen potential and the effectiveness of a vaccine containing the four antigens in providing cross-protective immunity against APEC. Our specific objectives were to 1) determine the proportion positive of selected antigens and their combinations among APEC isolates; 2) assess the ability of specific antigens to elicit IgY antibodies in chickens individually or in combination; 3) evaluate mRNA expression patterns of immune related genes in the bursa, spleen, and thymus in chickens vaccinated and challenged with APEC; 4) evaluate *in vitro* killing ability of serum and splenocytes from vaccinated chickens against multiple APEC serotypes; and 5) test protective ability of the vaccine against APEC challenge *in vivo*. This vaccine was developed to combat APEC infections in chickens with the goal of improving animal health and welfare.

## Results

### PCR screening reveals high proportion positive of *etsC*, *ompA*, *ompT*, and *traT* and their combinations among APEC isolates

Table 1 includes the proportion positive of *etsC*, *ompA*, *ompT*, and *traT* genes and their combinations in APEC isolates. Of the 80 strains tested, 98%, 84%, 73%, and 63% were positive for *ompT*, *ompA*, *etsC*, *and traT*, respectively. Most of the isolates (96%) had more than one of the four genes tested. Multiple combinations of these genes were found in the APEC isolates, the most common was the combination of the four genes *etsC ompA ompT traT* (45%), followed by *etsC ompA ompT* (16%), *ompA ompT* (12%), and *ompA ompT traT* (9%). The remaining combinations, including *etsC ompT*, *etsC ompT traT*, and *ompT traT* were less prevalent at 6%, 5%, and 3%, respectively (Table 1). We confirmed, using BLAST and PCR, that reference strains χ7122 (HE962388), APEC-O2 (CP006834), and MG1655 (CP014225) were positive for *etsC ompT traT*, *etsC ompT ompA traT*, and *ompT*, respectively.

**Table 1:** Prevalence of *etsC*, *ompA*, *ompT*, and *traT* genes and their combinations in APEC isolates and in specific APEC serotypes.

Genes/genotypes	% (Positive/total)	Serotypes (N)
**Genes**^a^		
*etsC*	73 (58/80)	O78 (16), NA (8), O1 (5), O45 (4), O11 (2), O115 (2), O131 (2), O2 (2), O22 (2), O71 (2), O83 (2), O138 (1), O15 (1), O153 (1), O173 (1), O18 (1), O21-O83 (1), O55 (1), O7 (1), O7-O157 (1), O8 (1).
*ompA*	84 (67/80)	O78 (16), NA (13), O45 (5), O1 (4), O22 (3), O55 (3), O115 (2), O131 (2), O18 (2), O2 (2), O10 (1), O114 (1), O138 (1), O15 (1), O153 (1), O173 (1), O23 (1), O6 (1), O7 (1), O7-O157 (1), O71 (1), O8 (1), O8-O60 (1), O83 (1), O9 (1).
*ompT*	98 (78/80)	O78 (19), NA (14), O1 (6), O45 (5), O131 (3), O22 (3), O55 (3), O11 (2), O115 (2), O2 (2), O71 (2), O83 (2), O10 (1), O14 (1), O138 (1), O15 (1), O153 (1), O173 (1), O18 (1), O21-O83 (1), O23 (1), O6 (1), O7 (1), O7-O157 (1), O8 (1), O8-O60 (1), O9 (1).
*traT*	63 (50/80)	O78 (12), NA (9), O1 (5), O45 (4), O22 (3), O11 (2), O131 (2), O2 (2), O55 (2), O115 (1), O138 (1), O15 (1), O23 (1), O7 (1), O71 (1), O8 (1), O8-O60 (1), O9 (1).
**Genotypes**^b^		
*etsC ompA ompT traT*	45 (36/80)	O78 (10), NA (6), O1 (4), O45 (4), O131 (2), O2 (2), O22 (2), O115 (1), O138 (1), O15 (1), O55 (1), O7 (1), O8 (1).
*etsC ompA ompT*	16 (13/80)	O78 (3), NA (2), O115 (1), O153 (1), O173 (1), O18 (1), O55 (1), O71 (1), O7-O157 (1), O83 (1).
*etsC ompT traT*	5 (4/80)	O11 (2), O71 (1), O78 (1).
*ompA ompT traT*	9 (7/80)	NA (2), O22 (1), O23 (1), O78 (1), O8-O60 (1), O9 (1).
*etsC ompT*	6 (5/80)	O78 (2), O1 (1), O21-O83 (1), O83 (1).
*ompA ompT*	13 (10/80)	NA (3), O78 (2), O10 (1), O114 (1), O45 (1), O55 (1), O6 (1).
*ompT traT*	3 (2/80)	NA (1), O1 (1).
*ompA*	1 (1/80)	O18 (1).
*ompT*	1 (1/80)	O131 (1).
*traT*	1 (1/80)	O55 (1).

^a^Gene present in any combination with other investigated genes; N, Number of strains;

^b^Combinations of genes. NA, not announced as serotype (not identifiable).

### Vaccination did not affect the growth of chickens

The average body weights of chickens on days 1, 14, and 28 post-vaccination are reported in Table 2. No significant differences were found between vaccinated and non-vaccinated chickens at any times tested.

**Table 2:** Body weight response to vaccination.

Treatment group	Body weight (g)		
	Day 1	Day 14	Day 28
**PBS**	54 ± 4	164 ± 14	332 ± 30
**Vaccinated**	58 ± 4	181 ± 17	331 ± 34

Data are depicted as mean ± SD in grams. No significant differences were detected within measurement day between treatment groups. N = 30 per group.

### Serum IgY antibody induction in response to vaccination

**Fig 1:**
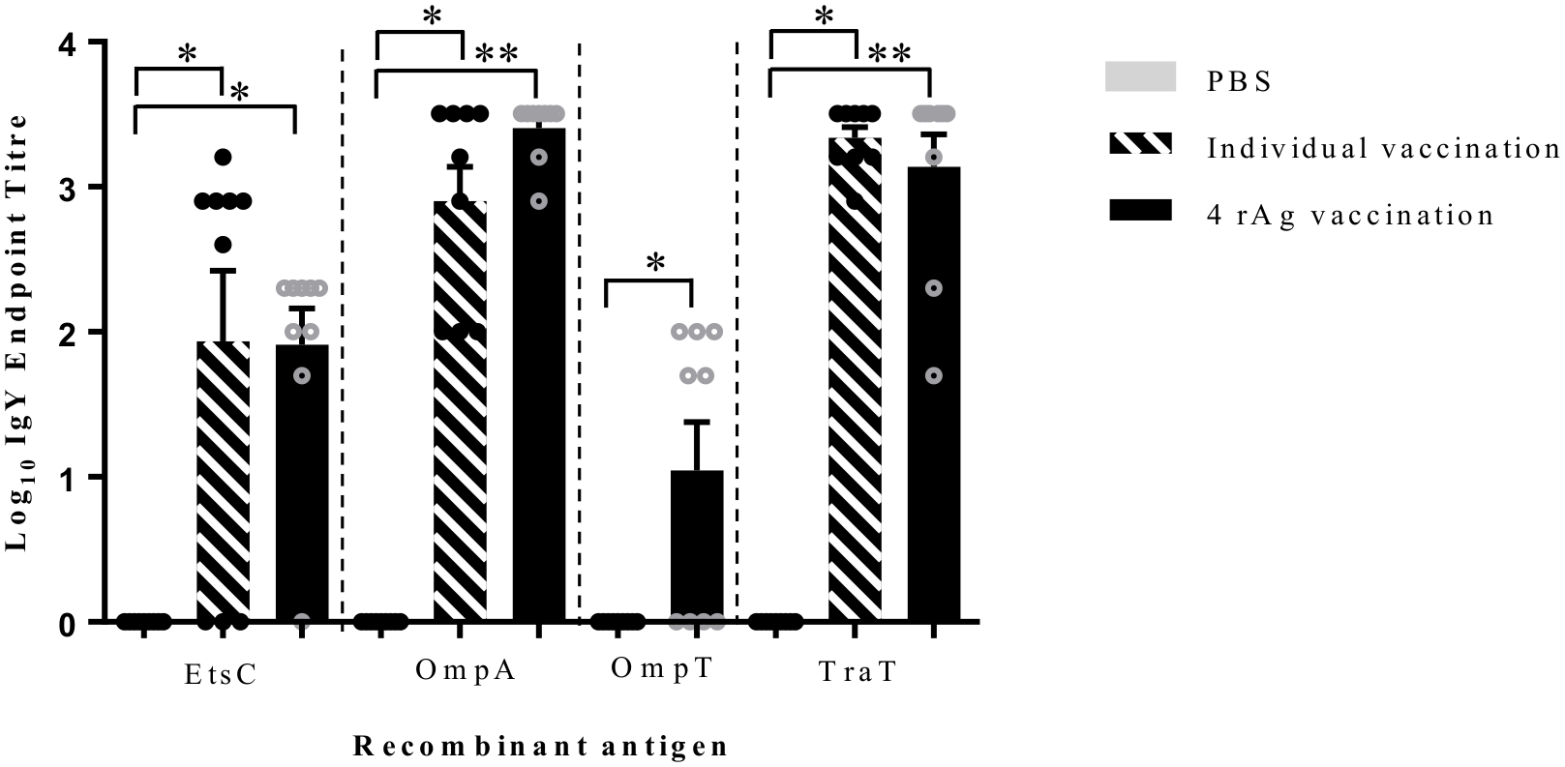
Serum IgY antibody response to vaccination. Data represents total serum IgY levels against individual recombinant antigens (rAg) induced in chickens vaccinated with either PBS (non-vaccinated), an individual antigen (EtsC, OmpA, or TraT), or with combined rAgs (EtsC, OmpA, OmpT, and TraT) in combination at day 29 post-vaccination. The serum from nine individual chickens/group were tested individually in duplicate. The values are shown as log_10_ IgY endpoint titer with mean ± SEM and individual animals are represented as dots. The grey bars are animals vaccinated with PBS (non-vaccinated), hashed bars represent animals vaccinated individually with EtsC, OmpA, or TraT (no individual vaccination was completed for OmpT), and black bars represent animals vaccinated with combined rAgs. Statistically significant differences were determined for each antigen compared to non-vaccinated by a Student’s T test, * indicates *P* < 0.05, and ** indicates *P* < 0.0001.

The levels of serum IgY antibody elicited in chickens vaccinated with EtsC, OmpA, OmpT, and TraT antigens individually or in combination as measured by ELISA at day 29 post first vaccination are depicted in Fig 1. All vaccinated chickens had significantly higher IgY antibody production than non-vaccinated chickens. Different antigens elicited different levels of IgY antibodies, and the order from highest to lowest signal was OmpA, TraT, EtsC, and OmpT. Administration of the antigens either alone or in combinations had no significant effect on the levels of specific antibodies elicited (Fig 1).

### Vaccination altered cytokines mRNA expression in vaccinated chickens challenged with APEC-O2

**Fig 2:**
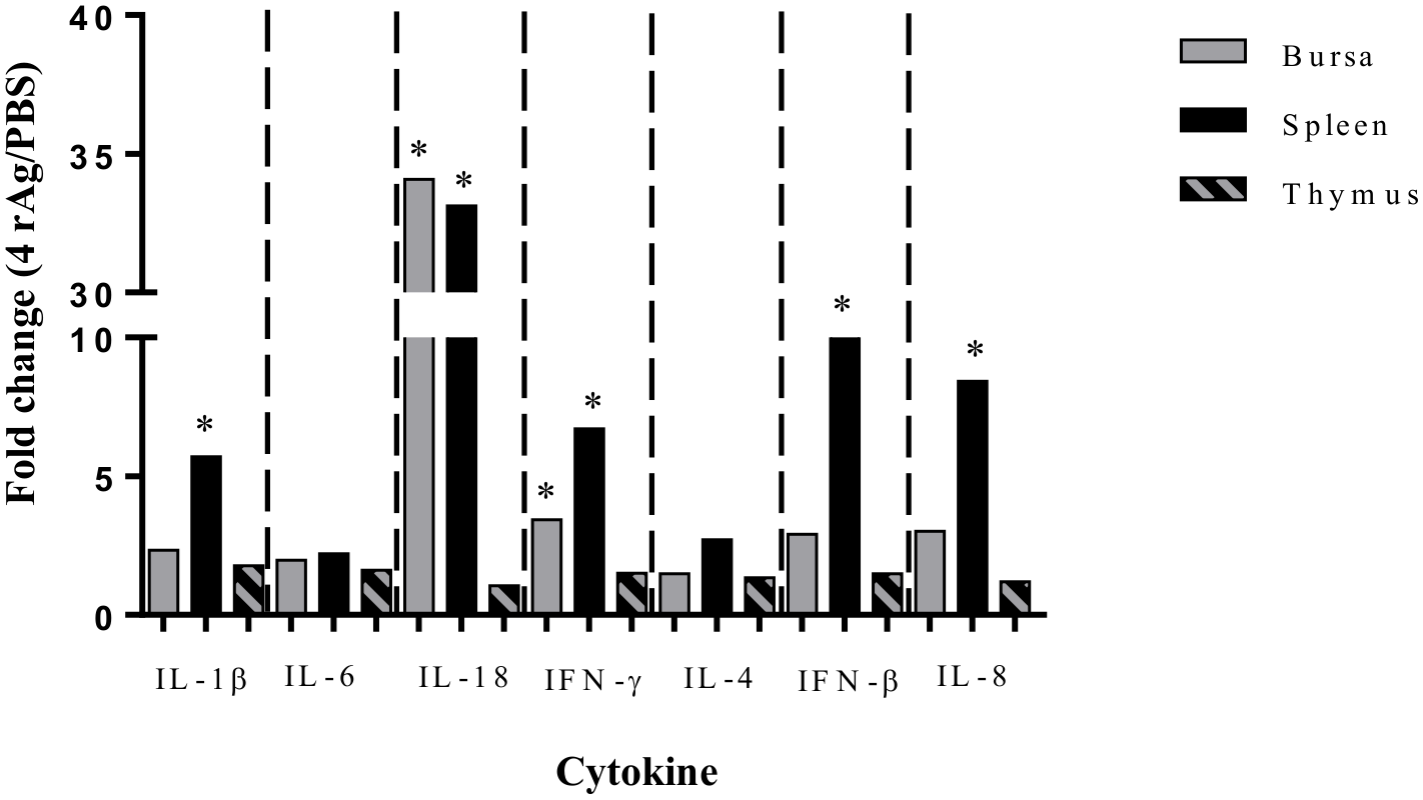
Changes in cytokine related gene expression in lymphoid organs of vaccinated chickens challenged with APEC-O2. Changes in IL-1β, IL-6, IL-18, IFN-γ, IL-4, IFN-β mRNA expression within the bursa, spleen, and thymus were determined by RT-qPCR. Expression levels are depicted as the fold change of chickens vaccinated with combined rAgs (EtsC, OmpA, OmpT, and TraT) that were challenged with APEC-O2, when compared to non-vaccinated (PBS) chickens that were challenged with APEC-O2. Bars show the fold change results from N = 5 chickens per tissue per group. The ddCt method was used to determine fold change using non-vaccinated as the reference i.e. a fold change >1 indicates increased expression levels due to vaccination. Differences within tissue between vaccinated and non-vaccinated chickens were considered significant at *P* ≤ 0.05 (*).

mRNA gene expression for IL-1β, IL-6, IL-18, IFN-γ, IL-4, IFN-β, and IL-8 in the bursa, spleen, and thymus shown as the fold change in vaccinated compared to non-vaccinated chickens that were challenged with APEC-O2 is shown in Fig 2. All genes showed upregulation in the vaccinated group compared to non-vaccinated. In the vaccinated chickens, the highest increases of significant mRNA expression changes were observed in the spleen (IL-1β, IL-18, IFN-γ, IFN-β, and IL-8), then in the bursa (IL-18 and IFN-γ), and no differences were observed in the mRNA gene expression of cytokines tested in thymus. The largest fold change (fold change > 32) in expression was identified for IL-18 in both the bursa and spleen tissues.

### Vaccination increased serum and splenocytes killing ability against multiple APEC serogroups

**Fig 3:**
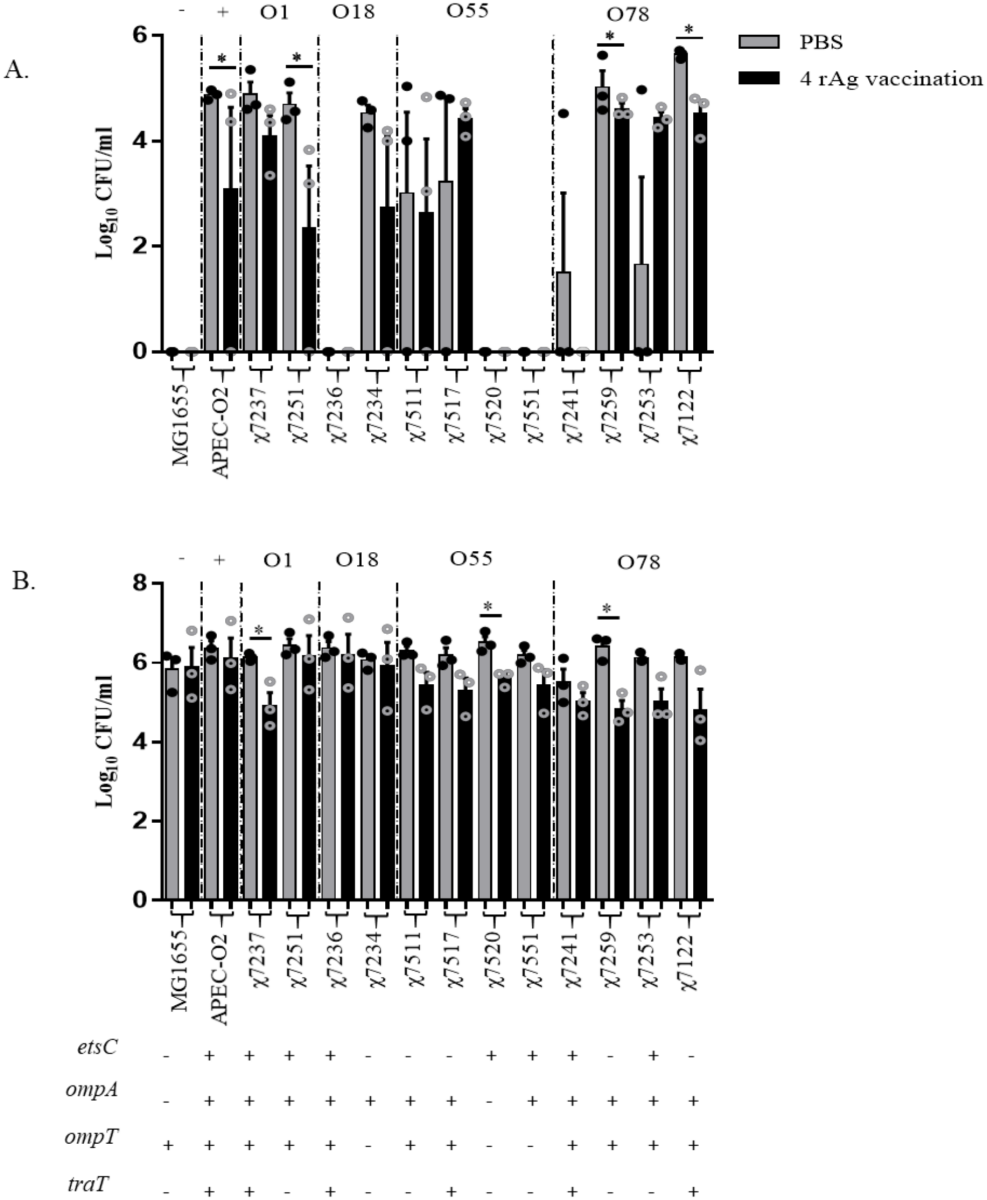
Bactericidal ability of vaccinated and non-vaccinated chicken serum and splenocytes. Chickens were vaccinated with either PBS (non-vaccinated) or with four rAgs (EtsC, OmpA, OmpT, and TraT). (**A**) Serum samples were collected from birds at 32 days post-vaccination and pooled (N = 10/treatment) within treatment group then tested for bacterial killing ability. Grey bars are non-vaccinated (PBS) and black bars are vaccinated (EtsC, OmpA, OmpT, and TraT). Complement sensitive MG1655 used as negative control (-), APEC-O2 used as positive control (+), and χ7122 was also used as positive control, and field APEC serogroups were tested (O1, O18, O55, and O78). (**B**) Spleen samples were collected from birds at 32 days post-vaccination and pooled (N = 5/treatment) within treatment group then tested for bacterial killing ability. The genotype of the strains determined by PCR as positive (+) or negative (-) for the genes *etsC*, *ompA*, *ompT*, and *traT*, is shown in the bottom of the graph. Samples were run in triplicate and the experiment repeated 3 times using independent pools. Dots represent individual samples from each experiment and bars are the mean ± SEM. Significant differences *P* < 0.05 calculated between vaccinated and non-vaccinated groups using Student’s T test with Tukey’s multiple testing correction.

The bactericidal activities of serum from chickens vaccinated with four antigens and those non-vaccinated were tested against the APEC isolates from the different serogroups, i.e. O1 (χ7237 and χ7251), O18 (χ7236 and χ7234), O55 (χ7511, χ7517, χ7520, and χ7551), and O78 (χ7241, χ7259, χ7253, and χ7122). The well characterized APEC-O1 (O1:K1), χ7122 (O78:K80), and non-pathogenic *E*. *coli* MG1655 strains were included (Table 3). Of the strains tested, APEC-O2, χ7237, and χ7241 contained all 4 antigens while the remainder of strains (except MG1655 and χ7234) contained some combination of the 4 antigens. In general bacteria grew better in serum from non-vaccinated than vaccinated chickens (Fig 3). As shown in Fig 3A, among fourteen strains tested, four strains, i.e. APEC-O2 (O2), χ7251 (O1), χ7259 (O78), and χ7122 (O78), had the most significant decreases in growth when exposed to serum from the vaccinated group compared to that of the non-vaccinated group, whereas all strains from serogroups O18 and O55 showed no significant differences between treatment groups. Three isolates, i.e. χ7236 (O18), χ7520 (O55), and χ7551 (O55), had little or no growth in both serum of vaccinated and non-vaccinated chickens, similar to the *E*. *coli* K-12 MG1655.

**Table 3:** Certain bacterial strains tested in this study.

Strain	Characteristics	Antigen genes*	Reference
**Reference strains**			
APEC-O2	APEC O2:K1:H7	*etsC ompA ompT traT*	[12]
χ7122	APEC O78:K80:H9, *gyrA*	*etsC ompT traT*	[23]
RS218	NMEC O18:H7:K1, ST95	*ompA ompT*	[24]
MG1655	Non pathogenic *E*. *coli* K-12	*ompT*	[25]
**Field isolates**			
χ7237	APEC O1	*etsC ompA ompT traT*	[26]
χ7251	APEC O1	*etsC ompA ompT*	[26]
χ7236	APEC O18	*etsC ompA ompT*	[26]
χ7234	APEC O18	*ompA*	[26]
χ7511	APEC O55	*ompA ompT*	[26]
χ7517	APEC O55	*ompA ompT traT*	[26]
χ7520	APEC O55	*etsC ompA ompT*	[26]
χ7551	APEC O55	*etsC ompA*	[26]
χ7241	APEC O78	*etsC ompA ompT traT*	[26]
χ7259	APEC O78	*ompA ompT*	[26]
χ7253	APEC O78	*etsC ompA ompT*	[26]

*genotype of field isolates was tested in this study; APEC, avian pathogenic *E*. *coli*; NMEC, neonatal meningitis *E*. *coli*.

Splenocytes were collected and tested against the same strains of APEC for their killing ability as in the serum bactericidal assay as shown in Fig 3B. Generally, the bacteria were killed at a fast rate by the splenocytes isolated from vaccinated group than by those from non-vaccinated one. The largest differences between treatment groups was identified in isolates of O1, O55, and O78 serogroups, i.e. χ7237 (O1), χ7520 (O55), and χ7259 (O78). No significant differences between treatments were identified in other strains, including the negative (MG1655) and positive (APEC-O2) controls.

### Vaccination with EtsC OmpA OmpT TraT protected chickens against APEC-O2 in an air sac challenge

**Fig 4:**
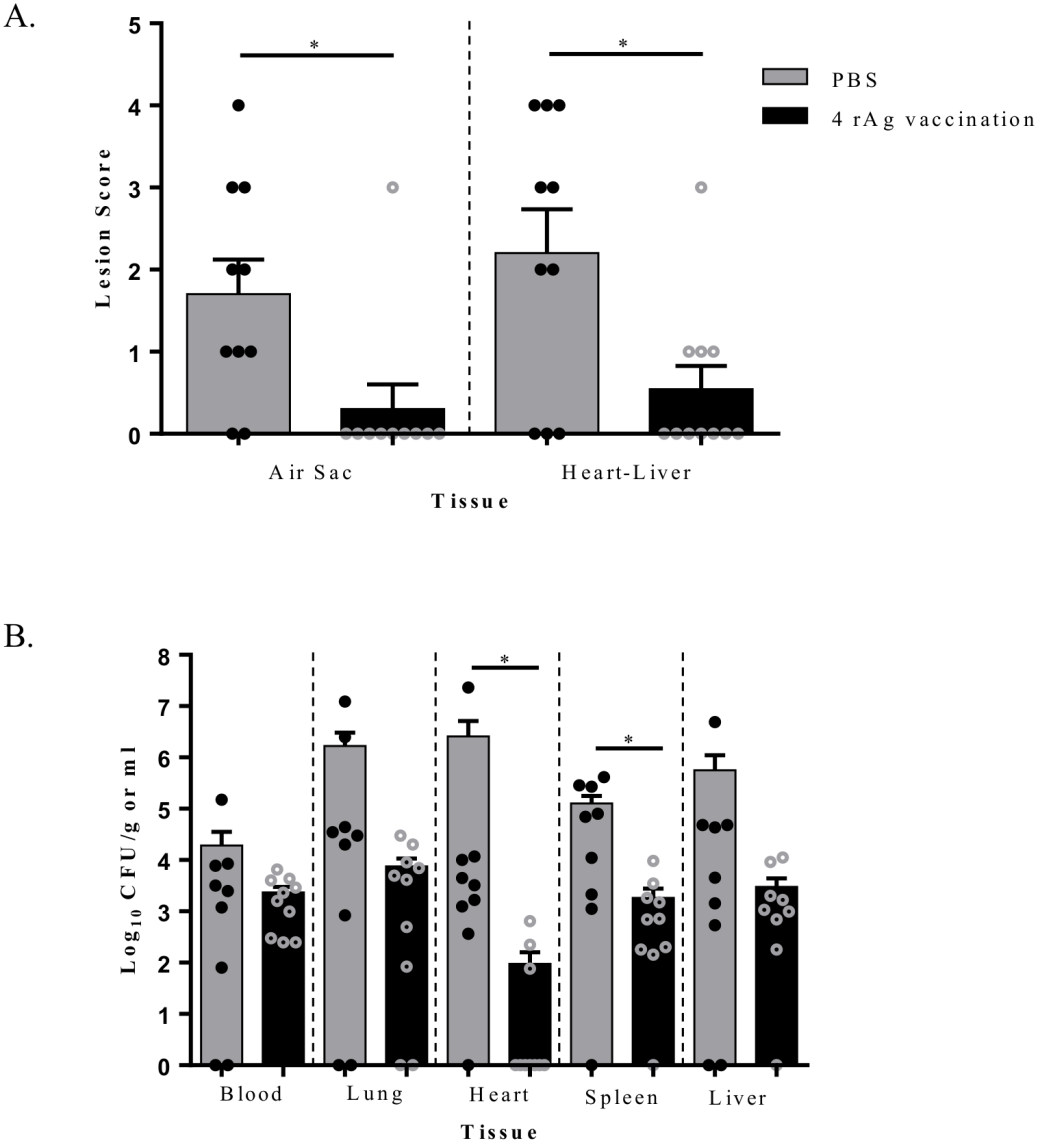
Bacterial loads in blood and organs of chickens challenged via air sac with APEC-O2. (**A**) Effect of vaccination on lesion scores in the air sac and heart-liver at 48 hpi (**B**) and on bacterial load in blood at 24 hpi and in organs (lung, heart, spleen, and liver) at 48 hpi. Chickens (N = 10) were vaccinated with either PBS (non-vaccinated) or combined rAgs (EtsC, OmpA, OmpT, and TraT) and then challenged with APEC-O2. Data are expressed as log CFU/g or ml. Significant *P* values in comparison to the non-vaccinated group were determined by Student’s T-Test within tissue (*P* < 0.05).

Lesion scores in air sacs and heart-liver and bacterial loads in blood and organs (lung, heart, spleen, and liver) of non-vaccinated and vaccinated chickens at 48 hours post infection (hpi) with APEC-O2 air sac-challenge are shown in Fig 4. Non-vaccinated chickens had higher levels of gross colibacillosis lesions (Fig 4A) and bacterial loads (Fig 4B) than vaccinated chickens. Vaccinated chickens showed significant (*P* < 0.05) reduction in air sac and heart-liver lesions. Most vaccinated chickens had no lesions in the air sac (9/10) and heart-liver (6/10); of 4 chickens that had lesions in the heart-liver, 3 had a minimal lesion score of 1. Whereas most non-vaccinated chickens had gross colibacillosis lesions in air sacs (8/10) and heart-liver (7/10).

Samples collected from vaccinated and non-vaccinated chickens at 24 hpi (blood) and 48 hpi (lung, heart, spleen, and liver) were enumerated for *E*. *coli*. As shown in Fig 4B, the vaccinated compared to non-vaccinated chickens had numerically lower CFU/g in blood and each tissue tested. The decrease in bacterial loads was significant (*P* < 0.05) in the heart and spleen, with the largest difference being in the heart. In the vaccinated group, few chickens (3/10) tested had bacteria in the heart and their levels were low (< 10^3^ CFU/g), while in the non-vaccinated group, most chickens (9/10) tested positive for *E*. *coli*, and their levels were between 10^2^ and 10^7^ CFU/g.

## Discussion

Vaccination capable of inducing cross-protection against the broadly distributed APEC serogroups is needed and would be highly demanded by poultry farmers. Previous studies suggested that multi-antigen vaccines would be more efficient in providing broad protection against the highly diverse ExPEC group [11, 27–29]. Here we developed and tested a novel vaccine containing four surface bacterial proteins EtsC, OmpA, OmpT, and TraT in chickens.

Previous works determined that *ompT*, *etsA*, and *etsB* genes were significantly more prevalent among disease causing *E*. *coli* compared to commensals [12, 30]. Additionally, EtsC, OmpA and TraT proteins were suggested as good vaccine targets due to their surface location and protein uniformity [31]. Here, PCR screening of eighty APEC isolates from colibacillosis cases collected in commercial production systems for the genes that encode the proteins used in our vaccine, i.e. *etsC*, *ompA*, *ompT*, and *traT*, confirmed the high proportion positive among APEC from diseased chickens [12, 30, 32]. In our isolates, these genes were found in different combinations, with the most prevalent being the four genes together across a wide variety of different serotypes, which justifies using these antigens in a vaccine against APEC. The APEC strains were from a wide range of serotypes (N = 26), reinforcing the notion of diversity of APEC found in the field which promotes the need for a cross protective vaccine.

Immunization with the rAgs EtsC, OmpA, OmpT, and TraT individually or in combination elicited detectable IgY antibodies against each antigen in the serum compared to non-immunized chickens. Detection of serum IgY antibodies against antigens tested indicate the potential of our vaccine to stimulate humoral immunity in chickens. Studies showed that when injected in mice, OmpA produced a substantial antibody response [33] and these antibodies protected against an *E*. *coli* sepsis challenge in mice [34]. Additionally, the elicitation of antibodies by TraT was previously tested in mice [35]. Here, we showed the elicitation of antibody by OmpT; the high variability (mean ± standard deviation is 1.04 ± 0.99) of serum IgY antibody elicited against OmpT, as compared to the other antigens tested, may be because the OmpT is not as efficiently recognized as the other antigens used in the current study by the immune system. To our knowledge, the antibody response to OmpT has never been reported previously and, our study is the first to test EtsC and OmpT antigens in a vaccine and no other report exists on serum IgY response in chickens to any of the reported proteins.

Interestingly, both plasmidic proteins (EtsC and TraT) elicited higher antibody levels than OmpT in chickens. Since EtsC and TraT are most associated with pathogenic bacteria, they could have been recognized more readily as foreign by the host than OmpT which is common among commensal *E*. *coli*. Future experiments could confirm this hypothesis, as well determine whether inclusion of EtsC, OmpA, and TraT without OmpT in a vaccine is sufficient to provide protection against APEC.

Previous studies have shown that TraT is a powerful antigen that can elicit strong immune responses in hosts without adjuvants added and could be used as a carrier molecule in vaccines [36–38]. Here, when injected individually, TraT did indeed elicit the strongest antibody response, but we have not tested the antigens without adjuvant, which could be performed in future studies.

It has previously shown that serum from chickens challenged with APEC O78 collected at 42 days post-challenge contained bactericidal ability against APEC O78 [39]. Here, to evaluate whether antibodies anti-antigens EtsC, OmpT, OmpA, and TraT elicited were broad protective, we tested the bactericidal effect of serum from vaccinated and non-vaccinated chickens against multiple APEC isolates and found that antibodies are protective against multiple APEC from different serogroups, including O1 and O78 commonly associated to avian colibacillosis [4]. These data indicate that our vaccine has broad protection potential and is not serogroup specific. The difference of susceptibility of the isolates from the same serogroup, i.e. O1, O18, O55, and O78 to the serum could be due to the difference of their genotype for the antigens genes but also the presence of capsules that could affect the accessibility of surface antigens.

Cytokine responses to vaccination and APEC challenged were evaluated in lymphoid organs, i.e. bursa, spleen, and thymus of chickens using RT-qPCR. Testing cytokines from different patterns, including proinflammatory (IL6, IL18, and IL1β), Th1 (IFNγ), Th2 (IL4), antiviral (IFNβ), and chemokine (IL8) is important to identify different immune pathways activated during vaccination and challenge. Previous studies identified gene expression in immune tissues in response to APEC challenge using high throughput methods [40–43]. In the current study, the ability of our vaccine to elicit increases (*P* < 0.05) in proinflammatory cytokines IL-1β and IL-18, a hallmark of APEC infection in poultry [39–41, 43], indicates the ability of our vaccine to activate pathways needed for effectively elimination of APEC. Our vaccine elicited also increased expression of IFNγ but not IL4, indicating a stronger Th1 cellular response. It is not clear why the vaccination did not increase the levels of IL-6 or IL-4 production in the spleen when tested at 48 hours post-challenge. IL-6 functions as a proinflammatory cytokine similarly to IL-1β, in which differences were detected. Future studies testing cytokines production at different time-points could detect their expression at earlier times post-challenge.

Gene expression in response to vaccination in combination with APEC challenge has only been described once with the rAg *Iss* vaccination, which had no effect on gene expression despite vaccine protection from gross lesions on internal organs [40]. However, Sandford et al., identified changes to the T cell receptor signaling pathway with upregulated genes belonging to clusters of differentiation molecules (ex. CD148 and CD45) in chickens challenged with APEC [40]. Testing other immune molecules in future studies would determine which other pathways could have been activated by our vaccine.

Among the lymphoid organ tested, the spleen responded most strongly to vaccination than the bursa and the thymus. Since the two later are intimately involved in the adaptive immune response such as antibody production, they could have been activated earlier, evaluation of cytokine responses at different time points of vaccination could show different cytokine expressions in different organs tested. Among the cytokines tested, the IL-18 gene had the strongest response (fold change >32) in both the bursa and spleen, indicating its importance in protection from vaccination during infection. In fact, IL-18 is a crucial proinflammatory cytokine involved in activation of both humoral and cellular immunity during microbial infections [44, 45], and has been shown to be an effective adjuvant for vaccines in chickens [46]. The IL-18 cytokine functions in chickens by stimulating the Th1 response by proliferation of CD4^+^ cells that are stimulated to release IFN-γ [47].

The spleen has an essential role in immune function. Splenocytes are a heterogeneous mixture of white blood cells that contains T and B lymphocytes, and phagocytes among others. Previous studies on chicken splenocytes have observed increased proliferation after vaccination with killed *Salmonella enteritidis* [48], which could correspond to increased bactericidal ability like in our study. Additionally, a study comparing bactericidal killing ability of splenocytes on *E*. *coli* identified differences between chickens infected with infectious Bronchitis Virus and non-infected ones [49], correlating functionality of splenocytes with immune status. Here splenocytes from vaccinated compared to non-vaccinated chickens were better able to kill APEC from different serogroups, i.e. O1, O55, and O78.

Strains tested had different sensitivity to killing ability of serum and splenocytes of vaccinated chickens, although the growth of APEC isolate χ7259 (O78) was reduced by both serum and splenocyte from vaccinated chickens, some strains were more sensitive to the serum than to the splenocytes, i.e. APEC-O2, χ7251 (O1), and χ7122 (O78); whereas it was the opposite for χ7237 (O1). Testing the sensitivity of multiple APEC strains to different immune mechanisms of defense activated by the vaccination, has determined that different APEC strains are combated differently by different host immune systems, however the ability of our vaccine to induce both humoral and cell-mediated immune responses, indicates its ability to target different APEC serotypes however providing broad protection.

We evaluated the vaccine protection using an i*n vivo* challenge with APEC-O2 that has all genes encoding the four proteins used in our vaccine, represents a common serogroup to cause colibacillosis in chickens, and has been fully described [12, 50, 51]. Chickens immunized with EtsC OmpA OmpT TraT had significantly reduced lesion scores in the air sac, heart, and liver, and bacterial loads in internal organs and blood in chickens, with the effect being significant in heart and spleen. This suggests that even with the invasive route of challenge, the vaccine could reduce infection. Additional studies could test protectivity to other serotypes *in vivo* and using a more natural route of infection such as intra-tracheal [52]. Here we evaluated the effect of vaccination on body weight, gross lesion, and internal organ bacterial enumerations. Future studies may include other measures to assess vaccine efficacy such as egg production, muscle mass accretion, and percent lethality post-challenge.

Combining all data together shows strong evidence for multifaceted protective vaccine including humoral, the mRNA regulation, cellular, and whole animal level. All antigens (EtsC, OmpA, OmpT, and TraT) were effective at inducing humoral immunity. The elicited antibodies could kill/reduce APEC from a wide variety of serogroups indicating one effective avenue of protection. The cytokine genes expression levels were most dramatically changed in the spleen due to vaccination, which lead us to investigate the cellular killing ability of splenocytes. Typically, APEC is transmitted via the respiratory system then it may enter the circulatory system. The spleen, a major contact point of host immune system with pathogens, is a critical tissue for clearance of pathogens. Our vaccine seems to be effective at killing APEC in firstly the blood, then any remaining pathogens may be killed partially within the spleen, indicating whole animal protection. This was demonstrated in the *in vivo* APEC challenge as chickens were partially protected from APEC-O2.

In summary, the multi-antigen EtsC OmpA OmpT TraT vaccine elicited a serum IgY response to each antigen in chickens and resulted in mRNA cytokines expression activation in the bursa and spleen. Broad protection potential against multiple APEC serotypes and decreased infection against APEC-O2 were demonstrated using an *in vitro* serum and splenocyte bactericidal assay, and a chicken APEC challenge, respectively. This study found that the multi-antigen vaccine could reduce the severity of APEC infection in chickens and could be applied for use in chickens to increase animal welfare and food production.

## Materials and methods

### Ethics statement

All animal experiments were conducted with approval from the Iowa State University Institutional Animal Care and Use Committee with log #1-16-8159-G. All animals were under the supervision of an attending veterinarian and procedures were used to reduce pain and distress. Animals received enrichments throughout the study and were acclimated to the animal facility for 3 days before handling or experimental treatment. The floor pen group housing system allowed socialization between animals. Animals were humanely euthanized in accordance with the American Veterinary Medical Association 2013 Guidelines using CO_2_ asphyxiation followed by secondary thoracotomy, either at specified experimental endpoints or upon recommendation of a veterinarian.

### Chickens

Ninety male and female specific-pathogen-free White Leghorn chickens were used in the study (VALO, Adel, IA). Animals had *ad libitum* access to water and feed (Purina organic grower feed) for the duration of the study, and were housed in floor pens with deep wood shavings. Animals were individually identified throughout the study using metal wing bands.

### Bacterial strains and PCR screening

Both reference *E*. *coli* strains and APEC isolates were used in the current study, some of them are listed in Table 3. The eighty APEC isolates were used to study the distribution of the *etsC*, *ompA*, *ompT*, and *traT* genes using PCR, were isolated from chickens and turkeys presenting signs of colibacillosis [26] and belong to 26 different serotypes. Of these, 11 isolates were used in bactericidal assays and are listed in Table 3. The reference APEC strain, APEC-O2 [51] was used in the *in vivo* challenge. Per BLAST, the genome of APEC-O2 (CP006834) contains *etsC*, *ompA*, *ompT*, and *traT* genes. The 80 isolates were PCR screened for the presence of the genes *etsC*, *ompA*, *ompT*, and *traT* using primer sequences listed in S1 Table. The positive controls included χ7122 for *etsC*, *ompT*, and *traT*, and RS218 for *ompA*.

Strains were usually grown in LB broth or agar containing 0.1% glucose overnight at 37°C. The challenge strain was grown in LB shaking to OD_600_ = 0.8, centrifuged at 4,000 X *g* for 20 min at room temperature, and the pellet was diluted to the appropriate concentration in PBS, and confirmed with serial dilutions plated on MacConkey agar and then incubated overnight at 37°C. MacConkey agar was also used to enumerate bacteria in serum, blood, and organs overnight at 37°C. All strains were stored in peptone-glycerol media at -80°C.

### Recombinant antigen preparation

The recombinant antigens were prepared as previously described [11]. Briefly, the genes that encode antigens EtsC, OmpA, OmpT, and TraT were PCR amplified from χ7122 (*etsC*, *ompT*, *traT*) and RS218 (*ompA*) strains using primers found in S1 Table, and then cloned into pET-101/D-TOPO^®^ vectors (Invitrogen). The sequence and the orientation of the genes in the plasmids were verified by sequencing. Recombinant proteins were produced in *E*. *coli* BL21, purified via His-tag using ProBond Ni-NTA resin columns (Invitrogen), and then endotoxin was removed using Pierce^™^ endotoxin removal spin columns (Thermo Scientific).

### Vaccination procedures

Chickens were injected subcutaneously with rAgs (EtsC, OmpA, OmpT, and TraT) in combination or individually (3 of 4 proteins were tested, i.e. EtsC, OmpA, and TraT) (Table 4). Since the focus is on the four antigens together, for individual antigen testing, we chose to test OmpA rather than OmpT due to its importance in virulence [16], its absence in the non-pathogenic *E*. *coli* MG1655 (Table 3), and to reduce the number of live animals used in the experiment. The rAgs were suspended in PBS containing 10 μg CpG (5’TCGTCGTTGTCGTTTTGTCGTT-3’) (Sigma-Aldrich) with a phosphorothioated backbone to increase the half-life *in vivo*, as an adjuvant. To prepare the vaccine the rAgs were diluted in PBS containing CpG to a final amount of 50 μg per rAg in a final volume of 200 μl for the 1^st^ vaccine on day 4 of age and boosted two weeks later with half the concentration of rAgs. Serum was collected at 29 days (for ELISA) and 36 days (for bactericidal assay) post first vaccination from coagulated blood and stored at -80°C until further analyses.

**Table 4:** Recombinant antigen concentration used in each vaccine dose.

Vaccination	Chicken age	Antigen concentration and volumes injected		
		Four antigens (EtsC OmpA OmpT TraT)	Individual antigens (EtsC, OmpA, or TraT)	Volume
**1^st^ vaccination**	4-day-old	50 μg/each (Total 200 μg)	50 μg/each (Total 50 μg)	200 μl
**2^nd^ vaccination**	18-day-old	25 μg/each (Total 100 μg)	25 μg/each (Total 25 μg)	200 μl

### IgY antibody response to vaccination in blood serum

Blood serum from all vaccine groups (N = 9/group) were tested for antibody response compared to the non-vaccinated (PBS) group collected on day 29 post-vaccination. Serum samples were diluted 1:200 in SEA blocking buffer and added to an ELISA plate coated with 2 μg/ml of individual rAg in coating buffer, serially diluted 1:2, and incubated for 1 hour at room temperature. Rabbit-anti-chicken-IgY-HRP antibody (Sigma) was used with the TMB substrate kit (Invitrogen). The relative antibody titer was determined as the reciprocal of the highest dilution that gave an absorbance at 450 nm twice of the control.

### RT-qPCR gene expression in spleen, bursa, and thymus

The bursa, spleen, and thymus from vaccinated and non-vaccinated chickens and challenged with APEC-O2 were aseptically collected. Samples were perforated and immediately put in RNAlater^™^ (Invitrogen) solution and kept at room temperature for 24 hours, then decanted and stored at -80°C until further use. Total RNA was isolated using PureLink^™^ Total RNA Purification (Ambion, Carlsbad, CA, USA) following manufacturer’s instructions, and stored at -80°C. RT-qPCR was performed using primers listed in S1 Table and Maxima SYBR Green/ROX qPCR kit (Thermo Scientific) with. Amplification and detection were completed using the StepOnePlus^™^ System (Applied Biosystems). For each treatment (vaccinated and non-vaccinated chickens) a total of 5 biological replicates were assayed in triplicate for the bursa, spleen, and thymus for each gene and assayed on the same qPCR plate. Samples were run with the following two-step conditions: 95°C for 10 min, 40 cycles of 95°C for 15 s, and 60°C 1 min. Fluorescence emission was recorded after each cycling step. Upon RT-PCR completion, melting curves were generated by increasing temperature from 60 to 95°C, followed by continued fluorescence acquisition.

### Serum bactericidal assay

A total of 11 APEC isolates were tested in the serum bactericidal assay. They belonged to serogroups O1, O18, O55, and O78 and had different combinations of selected genes (Table 1). Reference APEC strains, i.e. APEC-O2 and χ7122 (O78:K80), and non-pathogenic *E*. *coli* MG1655 were used as positive and negative controls, respectively.

Bacterial suspensions in PBS, prepared from overnight LB plates colonies were adjusted to OD_600_ of 0.1, were added into 90% of pooled serum from 10 individuals at a concentration of ~10^2^ CFU/100μl and incubated for 6 hours at 37°C. The rational for using undiluted serum with a concentration of 10^2^ bacteria was to attempt to replicate the *in vivo* conditions as closely as possible i.e. concentration of antibodies and approximate number of bacteria found in the blood during challenge (Fig 4B). Bacterial CFUs were determined by dilution and plating on MacConkey plates. All samples were run in duplicate and the experimental procedures were independently repeated 3 times.

### Splenocyte killing assay

Spleen cells were quickly thawed, washed 3 times in warm PBS. Cells within treatment group (vaccinated or non-vaccinated) were pooled (N = 5–7 per experimental replicate). Cells were separated with Histopaque-1077 with centrifugation at 400 x g for 30 minutes without brakes, the cloudy layer was collected, and washed 3 times with warm PBS. Cell viability was determined using trypan blue exclusion. Cells were suspended in RPMI-1640 with 10% FBS, and seeded at 10^5^ cells/well in 100 μl into 96 well cell culture plates at 41°C with 5% CO_2_. Cells adhered to plastic for 2 hours. A total of 11 APEC isolates were tested in the splenocyte assay. They belonged to serogroups O1, O18, O55, and O78 and had different combinations of selected genes (Table 1). Reference APEC strains, i.e. APEC-O2 and χ7122 (O78:K80), and non-pathogenic *E*. *coli* MG1655 were used as positive and negative controls, respectively. Bacterial suspensions in PBS, prepared from overnight LB plates colonies were adjusted to OD600 of 0.1. Bacteria were added to splenocytes at a MOI of 10:1 (bacteria:splenocyte). Plates were centrifuged at 1,000 x g, and incubated for 1 hour at 41°C with 5% CO_2_. Cells were washed twice with warm PBS, and then lysed with 1% sodium deoxycholate for 10 minutes at room temperature. Bacterial CFUs were determined by dilution and plating on MacConkey agar plates. All samples were run in duplicate and the experimental procedures were independently repeated 3 times.

### Chicken air sac challenge with APEC-O2

On day 30 post-vaccination, chickens were inoculated with ~5x10^8^ CFU (100 μl of PBS) of APEC-O2 via intra-air sac. Chickens were monitored twice daily, and euthanized using CO_2_ asphyxiation at 48 hpi. Lesion scoring for inflammation and gross lesions on the air sac [0, normal; 1, slight edema; 2, mild diffuse thickening and neovascularization of air sacs with mild fibrinous exudate; 3, moderate fibrinous exudate; 4, severe extensive exudate], and combined lesion scoring for heart and pericardium [0, normal; 1, vascularization, opacity, cloudy fluid in the pericardial cavity; 2, acute pericarditis], and liver [0, normal; 1, slight amounts of fibrinous exudate; 2, marked perihepatitis] were performed [53]. Chicken samples were aseptically collected from either live chickens at 24 hpi (blood) or euthanized chickens at 48 hpi (lung, heart, spleen, and liver). Tissues were weighed, diluted in PBS, and serial dilutions were plated on MacConkey agar and incubated overnight at 37°C. The CFUs per g or ml were calculated.

### Statistical analyses

All statistical analyses were run in GraphPad Prism software. Each vaccinated group versus non-vaccinated (PBS) control were compared using Student’s T-test. For ELISA and serum bactericidal assays, only duplicate samples within 20% of their mean were used. Means were compared (N = 9-10/group) to the non-vaccinated group for each assay. For the serum bactericidal assay, the mean of three independent experimental replicates were determined within group and compared to non-vaccinated chicken serum using Student’s T-test. *P* values < 0.05 were considered significant. To determine the relative gene expression, the ddCt method was used [54]. Delta Ct values were obtained by normalizing the Ct values of the target genes with the reference gene 28S. Fold induction of the gene expression was estimated as 2^-ddCt^. Non-vaccinated (control) samples were used as calibrators. The statistical analyses for RT-PCR were completed using the delta-delta Ct values comparing vaccinated to non-vaccinated using Student’s T-test and *P* values < 0.05 were considered significant.

## Supporting information

S1 Table Primers used in the study. PCR primers for amplification of *etsC*, *ompA*, *ompT*, and *traT* to produce recombinant antigens, PCR primers for screening of field APEC strains, and RT-PCR primers for mRNA gene expression.
(DOCX)
