# Supplementary material for: A recombinant multi-antigen vaccine with broad protection potential against avian pathogenic Escherichia coli
Source: PLoS One. 2017 Aug 24;12(8):e0183929. doi: 10.1371/journal.pone.0183929 (PMC5570496; doi:10.1371/journal.pone.0183929)
Supplement: S1 Table — PCR primers for amplification of etsC, ompA, ompT, and traT to produce recombinant antigens, PCR primers for screening of field APEC strains, and RT-PCR primers for mRNA gene expression. (DOCX) [file pone.0183929.s001.docx]

**S1 Table**

| Gene Primer (5’ to 3’) Amplicon (bp) Reference | | | |
| --- | --- | --- | --- |
| Recombinant antigen cloning | | | |
| *etsC* | F: CGCGGATCCGTTTATGCCTTATCACCGC | 1,352 | This study |
|  | R: CCCAAGCTTGATTAAGGAAAGGTTGATGCC |  |  |
| *ompA* | F: CACCATGAAAAAGACGCT | 1,042 | This study |
|  | R: AGCCTGCGGCTGAGTTAAAC |  |  |
| *ompT* | F: CACCATGTACTTAAAGATTCTTGC | 956 | This study |
|  | R: AAAATAATACTTCAGACCAGCTGTGGT |  |  |
| *traT* | F: CGCGGATCCGGATAAGAAAACT | 778 | This study |
|  | R: GAGAATATTTGCGATTGATTTGGC |  |  |
| PCR screening of field APEC strains | | | |
| *etsC* | F: GGATGCGGAAAGAACAGGTA | 202 | [1] |
|  | R: TTCTTCACTGGCATGGACTG |  |  |
| *ompA* | F: AGCTATCGCGATTGCAGTG | 919 | [2] |
|  | R: GGTGTTGCCAGTAACCGG |  |  |
| *ompT* | F: CCTCCACGACCAGCTAATGT | 196 | [2] |
|  | R: CGGAGATTGATTTTGGCACT |  |  |
| *traT* | F: GGTGTGGTGCGATGAGCACAG | 290 | [2] |
|  | R: CACGGTTCAGCCATCCCTGAG |  |  |
| RT-PCR for mRNA gene expression NCBI gene ID | | | |
| *IL-1β* | F: GGAGGTTTTTGAGCCCGTC | 395196 | [3] |
|  | R: TCGAAGATGTCGAAGGACTG |  |  |
| *IL-6* | F: AGGACGAGATGTGCAAGAAGTTC | 395337 | [4] |
|  | R: TTGGGCAGGTTGAGGTTGTT |  |  |
| *IL-18* | F: GAAACGTCAATAGCCAGTTGC | 395312 | [5] |
|  | R: TCCCATGCTCTTTCTCACAACA |  |  |
| *IFN-γ* | F: ACACTGACAAGTCAAAGCCGC | 396054 | [5] |
|  | R: AGTCGTTCATCGGGAGCTTG |  |  |
| *IL-4* | F: GCTCTCAGTGCCGCTGATG | 416330 | [6] |
|  | R: GGAAACCTCTCCCTGGATGTC |  |  |
| *IFN-β* | F: ACCAGATCCAGCATTACATCCA | 554219 | [6] |
|  | R: CGCGTGCCTTGGTTTACG |  |  |
| *IL-8* | F: AAGGATGGAAGAGAGGTGTGCTT | 396495 | [6] |
|  | R : GCTGAGCCTTGGCCATAAGT |  |  |
| *28S* | F: GGCGAAGCCAGAGGAAACT | 107049346 | [6] |
|  | R : GACGACCGATTTGCACGTC |  |  |

1. Ewers C, Li G, Wilking H, Kiessling S, Alt K, Antáo EM, et al. Avian pathogenic, uropathogenic, and newborn meningitis-causing *Escherichia coli*: how closely related are they? Int J Med Microbiol. 2007;297(3):163-76.

2. Mellata M, Ameiss K, Mo H, Curtiss R. Characterization of the contribution to virulence of three large plasmids of avian pathogenic *Escherichia coli* chi7122 (O78:K80:H9). Infect Immun. 2010;78(4):1528-41.

3. Dunislawska A, Slawinska A, Stadnicka K, Bednarczyk M, Gulewicz P, Jozefiak D, et al. Synbiotics for Broiler Chickens-In Vitro Design and Evaluation of the Influence on Host and Selected Microbiota Populations following In Ovo Delivery. PLoS One. 2017;12(1):e0168587.

4. Chiang HI, Berghman LR, Zhou H. Inhibition of NF-kB 1 (NF-kBp50) by RNA interference in chicken macrophage HD11 cell line challenged with *Salmonella enteritidis*. Genet Mol Biol. 2009;32(3):507-15.

5. Brisbin JT, Gong J, Parvizi P, Sharif S. Effects of lactobacilli on cytokine expression by chicken spleen and cecal tonsil cells. Clin Vaccine Immunol. 2010;17(9):1337-43.

6. Sławinska A, Siwek MZ, Bednarczyk MF. Effects of synbiotics injected in ovo on regulation of immune-related gene expression in adult chickens. Am J Vet Res. 2014;75(11):997-1003.
